# The Effect of Suppressed Levels of Uninvolved Immunoglobulins on the Prognosis of Symptomatic Multiple Myeloma

**DOI:** 10.4274/tjh.2016.0161

**Published:** 2017-06-01

**Authors:** Murat Sarı, Selma Sarı, Meliha Nalçacı

**Affiliations:** 1 İstanbul University İstanbul Faculty of Medicine, Department of Internal Medicine, İstanbul, Turkey

**Keywords:** Multiple myeloma, Prognostic factors, Serum immunoglobulins, Nephelometric measurement, M-protein

## Abstract

**Objective:**

The majority of multiple myeloma (MM) patients have high levels of monoclonal immunoglobulin in the serum and/or urine and suppressed levels of the uninvolved immunoglobulins. The prognostic significance of this phenomenon has not been assessed sufficiently. In this study, our aim is to evaluate the prognostic significance of uninvolved immunoglobulin suppression measured by nephelometry in patients with new symptomatic MM and the association with other features of the disease.

**Materials and Methods:**

Between August 2003 and February 2015, 137 patients who were referred for the treatment of newly diagnosed symptomatic myeloma to the Hematology Department polyclinics of the İstanbul University İstanbul Faculty of Medicine were prospectively included and had available pretreatment immunoglobulin levels measured by nephelometry.

**Results:**

Suppression of at least one uninvolved immunoglobulin was observed in 87% of patients and this situation was slightly more common in patients with immunoglobulin A myeloma but had no statistical significance (p>0.05). Uninvolved immunoglobulin suppression was also more common among patients who had bone marrow plasma cell infiltration of ≥40% and presented with anemia and hypercalcemia (p<0.05). The overall survival time was shorter in patients with positive calcium-renal-anemia-bone criteria and International Staging System stage 3 compared with others (p<0.05). Factors that were independently associated with inferior survival in the multivariate analysis included patients with estimated glomerular filtration rate of <60 mL/min, age of >65 years, lactate dehydrogenase of >300 IU/L, bone marrow plasma cells of ≥40%, and β2-microglobulin of >3.5 mg/dL (p<0.05).

**Conclusion:**

In this study, 13.1% of MM patients had preserved levels of uninvolved immunoglobulins. We observed that patients who had preserved uninvolved immunoglobulin levels had better treatment responses and better pathologic signs, but statistical significance could not be shown. Conversely, patients with suppression of even one of the uninvolved immunoglobulins had a shorter survival, but similarly, statistical significance could not be shown.

## Abstract (tr)

**Amaç:**

Multipl miyelom (MM) tanılı hastaların büyük çoğunluğunda serum ve/veya idrarda yüksek seviyelerde saptanan monoklonal immünglobulinler mevcut iken hastalıkla ilişkili olmayan immünglobulin düzeylerinde ise baskılanma mevcuttur. MM’de bu fenomenin prognostik önemi yeteri kadar değerlendirilmemiştir. Biz bu çalışmada, nefelometrik ölçümler ile belirlenmiş, hastalıkla ilişkili olmayan immünglobulin düzeylerindeki baskılanmanın yeni tanı semptomatik MM hastalarında prognoz ve hastalığın diğer özellikleri ile ilişkisini incelemeyi amaçladık.

**Gereç ve Yöntemler:**

Ağustos 2003 ve Şubat 2015 tarihleri arasında İstanbul Üniversitesi İstanbul Tıp Fakültesi, İç Hastalıkları Anabilim Dalı, Hematoloji Bilim Dalı Polikliniği’ne yeni semptomatik miyelom tanısı ile başvuran 137 hasta retrospektif olarak çalışmaya alındı. Bu hastaların hepsinde tedavi öncesi nefelometrik yöntemler ile bakılmış immünglobulin düzeyleri mevcuttu.

**Bulgular:**

Hastaların %87’sinde hastalıkla ilişkili olmayan immünglobulin düzeylerinden en az birinde baskılanma mevcuttu ve immünglobulin A miyelom tanılı hastalarda istatistiksel olarak anlamlı olduğu saptanamasa da bu durum daha sık görüldü. Kemik iliği plazma hücre infiltrasyonu ≥%40 bulunan, anemi ve hiperkalsemi ile başvuran hastalarda hastalıkla ilişkili olmayan immünglobulin düzeylerinde baskılanma daha sık görüldü (p<0,05). Hiperkalsemi, böbrek yetmezliği, anemi, kemik hastalığı kriterleri pozitif olan ve Uluslararası Evreleme Sistemi evre 3 hastalığı olan hastaların diğerlerine göre genel sağkalımı daha kısa idi (p<0,05). Çoklu değişkenli analizler sonucunda; tahmini glomerüler filtrasyon hızı <60 mL/dk, yaş >65, laktat dehidrogenaz >300 IU/L, kemik iliği plazma hücre infiltrasyon oranı ≥%40 ve β2-mikroglobulin >3,5 mg/dL gibi faktörlerin sağkalım açısından olumsuz birer faktörler olduğu ortaya çıktı (p<0,05).

**Sonuç:**

Bu çalışmada hastaların %13,1’inde immünglobulin düzeylerinin korunmuş olduğu görüldü. İmmünglobulin düzeyi korunmuş hastaların daha iyi tedaviye yanıt oranlarına ve daha iyi patolojik bulgulara sahip olduğu görüldü ama immünglobulin düzey korunmasının hastalar açısından bağımsız olumlu bir prognostik faktör olduğu gösterilemedi. Aynı şekilde immünglobulin baskılanması olan hastaların yaşam süresi daha kısa bulundu, ancak bu da bağımsız olumsuz bir risk faktörü olarak belirlenemedi.

## INTRODUCTION

Excessive amounts of a monoclonal immunoglobulin (Ig) or parts of Igs are produced and secreted in multiple myeloma (MM) [1]. Around 97% of patients with MM have high levels of these monoclonal proteins (M-proteins), which can be detected through protein electrophoresis in serum and/or urine. Quantification of Igs with nephelometry is extensively used and has been validated as a method of assessment of Ig levels. Current studies have shown that suppression of uninvolved Igs in monoclonal gammopathy of undetermined significance (MGUS) and smoldering MM increases the risk of progression to symptomatic MM [2,3,4,5]. However, there are limited data and studies on the prognostic significance of uninvolved Ig suppression in patients with symptomatic MM. Lately, novel immunoassays that measure serum concentrations of the Ig heavy-chain/light-chain subsets IgG kappa, IgG lambda, IgA kappa, and IgA lambda have been developed; this method identifies suppression of uninvolved Ig of the same isotype as the tumor and shows prognostic significance in patients MM [6]. However, this method is not widely available yet.

In our study, we aimed to assess the prognostic significance and association of the suppression of uninvolved Igs with other features of the disease, as measured using nephelometry.

## MATERIALS AND METHODS

### Patients and Follow-Up

Between August 2003 and February 2015, 137 patients who were referred to the Hematology Department polyclinics of the İstanbul University İstanbul Faculty of Medicine for the treatment of newly diagnosed symptomatic myeloma were prospectively included and had available pretreatment (before initiation of any antimyeloma therapy and within 1 month of the diagnosis of MM) Ig levels measured by nephelometry. Biochemical tests of patients were conducted at the Central Clinical Biochemistry Laboratory of the İstanbul University İstanbul Faculty of Medicine Hospital (Roche Modular P chemistry analyzer). In this retrospective analysis, which includes a 12-year period, only cut-off values of lactate dehydrogenase (LDH) were changed due to changing of the analyzer (before reference values were 230-420 IU/L; now they are 135-250 IU/L). Some patients were treated with classic chemotherapy as a first-line therapy. Vincristine, adriamycin, and dexamethasone or melphalan-prednisolone regimens were used as classical chemotherapy. The majority of the patients were treated with novel agents that were bortezomib-based regimens as a first-line therapy. Deceased patients were identified using the Republic of Turkey Ministry of Health Mortality Reporting System, outpatient file records, and the epicrisis, and final statuses of surviving patients were confirmed by phone.

Suppression of Igs was defined as a reduction of an uninvolved Ig (IgM and IgA levels in the case of IgG myeloma) below the lower normal limit, which for IgG was <700 mg/dL, for IgA was <70 mg/dL, and for IgM was <40 mg/dL.

Renal function was assessed using the estimated glomerular filtration rate (eGFR), which was calculated using the modified Modification of Diet in Renal Disease formula and serum creatinine levels. Hypercalcemia was defined as a corrected serum calcium level of ≥11.5 mg/dL, and Hb of <10g/dL was accepted as anemia.

The International Myeloma Working Group criteria were used for assessing response to treatment [7]. For the purpose of the current analysis, complete response was defined as confirmed negative immunofixation of serum and urine. Very good partial response included patients with ≥90% reduction of the M-spike and urine M-spike of <100 mg/day, as well as patients with no paraprotein in serum or urine electrophoresis but with positive immunofixation. A partial response was defined as a ≥50% decrease in serum M-protein concentration and a ≥90% decrease in urine M-protein excretion.

### Statistical Analysis

SPSS 17.0 was used for the statistical analysis of this study. Comparisons for categorical variables among different groups were made using the chi-square test and Fisher’s exact test when appropriate. Overall survival (OS) was measured from the date of treatment initiation until the date of death or date of last follow-up. Progression-free survival (PFS) was calculated from the date of initiation of therapy until the date of the first evidence of disease progression or death. Patients without evidence of progressive disease were censored at the date of last follow-up. Time to the realization of the event showed the treatment results (time-to-event), Kaplan-Meier curves were plotted, and comparisons among groups were made using the log-rank test. For the multivariate analysis, factors associated with time-to-event were introduced into a Cox proportional hazards model. Values of p<0.05 were considered statistically significant.

## RESULTS

We evaluated 137 patients with newly diagnosed symptomatic MM. Table 1 shows the disease features of the patients with suppressed uninvolved Igs and of those with preserved uninvolved Igs. The median age of the whole study group was 62 years (range: 26-97 years). Suppression of at least one uninvolved Ig was observed in 87% of patients (only 13% of all patients had a preserved uninvolved Ig), and at least two suppressed uninvolved Igs were found in 69% of the patients. Suppression of at least one uninvolved Ig was slightly more common in patients with IgA myeloma (96% vs. 83.7% for IgG myeloma and 95.2% for light-chain myeloma, p>0.05).

Anemia (hemoglobin of <10 g/dL; p<0.05), hypercalcemia (Ca of ≥11.5 mg/dL; p<0.05), and osteolytic bone disease (p<0.05) were more frequent in patients with suppressed uninvolved Igs, as shown in Table 1. Renal dysfunction (eGFR of <60 mL/min per 1.73 m2) was seen in 45% of patients and was more commonly seen in patients with suppressed uninvolved Igs, albeit without a significant relationship (p>0.05). Suppression of the uninvolved Igs was more frequent in patients with extensive bone marrow infiltration (p<0.001). Furthermore, 31% of patients had International Staging System (ISS) stage 1, 28% had ISS stage 2, and 41% of patients had ISS stage 3 disease. Although advanced-stage disease was more commonly seen in patients with suppressed uninvolved Ig (at least one was suppressed in 85.7% vs. 84.2% vs. 89.5% of patients with ISS-1, -2, and -3 disease, respectively, p>0.05), no statistically significant relationship was detected.

Ninety-two percent of all patients had at least one positive calcium-renal-anemia-bone (CRAB) criterion met. One, 2, 3, or 4 positive CRAB criteria were seen more commonly in patients with at least two Igs suppressed and this was statistically significant (p<0.001). In addition, CRAB criteria were seen less commonly in those with uninvolved preserved Igs; 3 or 4 positive CRAB criteria were also not seen in this group (p<0.05).

Thirty-five percent of all patients (n=48) had a complete response to treatment, 17.5% (n=24) had a very good partial response, 35.8% (n=49) had a partial response, and 11.7% (n=16) did not respond to treatment. Eighty-eight percent of all patients had a partial or higher response to treatment. Although groups with preserved uninvolved Igs had partial responses or better to treatment, statistical significance could not be seen (97.4% versus 88% and 87.2%, p>0.05).

Twenty percent of all patients received conventional chemotherapy, 4% had conventional chemotherapy and autologous stem cell transplantation, 58% received new-generation novel agents, and 18% had new-generation novel agents and autologous stem cell transplantation in the form of first-line therapy.

Clinical recurrence was seen in 25 (20.6%) patients, 11 of whom died; the remaining 14 patients were still living (14/84 patients). The median follow-up time for living patients without relapse was 48.6 months (range: 40.2-57.1 months). Relapsed patients were more commonly from the group with at least two uninvolved Igs suppressed (19 patients). However, there was no statistically significant relationship between relapse and uninvolved Ig suppression. Although relapse and death were more commonly seen in the suppressed uninvolved Ig group, statistical significance was not seen.

Death occurred in a total of 53 patients. The median OS time was 76 months (range: 44.2-106 months). Diagnosis and the first 6 months of treatment are very important for survival. The death rate and survival rate was 3% and 97% in the first 3 months, respectively. After 6 months, the death rate was 6% and the cumulative survival rate was 94%. Thirteen deaths occurred among 137 patients in the 1-year follow-up period. The mortality rate was 10% and the cumulative survival rate was 90%. The cumulative survival rate fell to 82% at 2 years, 70% at 3 years, 55% at 5 years, and 30% at 10 years.

**Figure 1:**
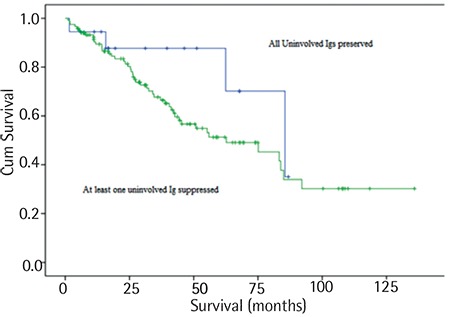
Kaplan-Meier survival estimate regarding overall survival for patients with preserved immunoglobulins and for patients with suppressed immunoglobulins.

The median OS was 85.5 months for patients with preserved uninvolved Igs and was 62.6 months for patients with at least one Ig suppressed; however, no statistical significance could be detected, as shown in Figure 1. The median OS time was shorter among patients with at least two uninvolved Ig suppressed than in the other patients (55.2 months; range: 36.2-74.1 months).

**Figure 2:**
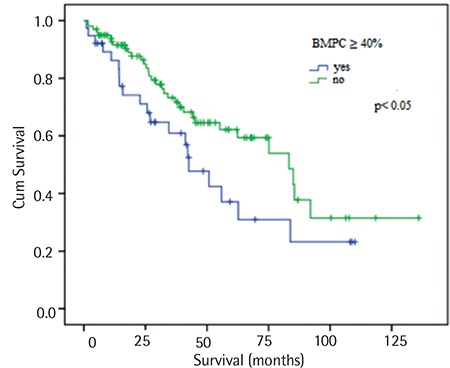
Kaplan-Meier survival estimate regarding overall survival for patients with bone marrow plasma cells of ≥40%. BMPC: Bone marrow plasma cell.

**Figure 3:**
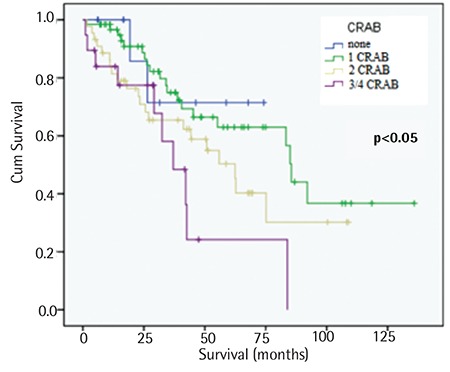
Kaplan-Meier survival estimate regarding overall survival for patients with calcium-renal-anemia-bone criteria. CRAB: Calcium-renal-anemia-bone.

Patients with bone marrow plasma cells (BMPCs) of ≥40% had a median survival time shorter than the other patients (42.6 vs. 83.2 months, p<0.05), as shown in Figure 2. The OS time was shorter in patients with positive CRAB criteria than in patients without CRAB criteria. There was a significantly shorter OS time in patients with 3 and 4 positive CRAB criteria than in the other patients (37 months, p<0.05), as shown in Figure 3.

OS was significantly shorter in patients with ISS stage 3 compared with other stages (42 months vs. 83.2 and 84.9 months). There was a significant difference in terms of survival between Ig levels of preservation and suppression and ISS stage 1 and stage 3 disease (p<0.05). The median PFS of all patients was 41.2 months (range: 34-48.4 months). The median PFS of patients with preserved uninvolved Ig was 63.1 months (range: 46.5-79.6 months), and patients with at least one uninvolved Ig suppressed had 38.8 months of PFS time (range: 29.4-48.1 months) (p>0.05).

We performed a multivariate analysis to adjust for the impact of uninvolved Ig suppression and other well-defined prognostic factors on survival. We found no significant relationship between uninvolved Ig suppression or preservation and survival. Other factors that were independently associated with inferior survival in the multivariate analysis included ISS stage 3 disease with 2.76 times greater periodic risk of death than stage 1 disease [95% confident interval (CI): 1.36 to 5.59], while patients with eGFR of <60 mL/min had 2.28 times greater periodic risk (95% CI: 1.32-3.95) and patients aged >65 years had 2.11 times greater periodic risk (95% CI: 1.23-3.63). Patients with LDH of <300 IU/L had a 2.5 times reduced periodic risk of death. Due to change of analyzer, previously obtained values of >300 IU/L with the other analyzer were considered normal in the statistical analysis, but despite this LDH of >300 IU/L still appeared to be a poor prognostic factor. Again, BMPCs below 40% were associated with a 2-fold reduction in terms of risk of death. For patients with β2-microglobulin of <3.5 mg/dL, periodic risk of death was reduced by 2.5 times, as summarized in Table 2.

## DISCUSSION

In this study, we aimed to assess the prognostic significance and association of the suppression of uninvolved Igs with other features of the disease, as measured using nephelometry. Only 13% of all patients had preserved uninvolved Ig levels and the rest of the patients (87%, 119 of 137 patients) had suppression of at least one uninvolved Ig. Suppression of at least one uninvolved Ig was slightly more common in patients with IgA myeloma (96% vs. 83.7% for IgG myeloma and 95.2% for light-chain myeloma; p>0.05). Our results were similar to the report of Kyle et al. [1] with a large cohort of 1027 patients with symptomatic MM from the mayo clinic and the results of the study by Kastritis et al. [8], which consisted of 1755 patients, but we found no statistically significant relationship between the type of MM and suppression of uninvolved Ig levels. In terms of clinical features of MM, response rates to treatment, and survival rates, we saw that the preserved uninvolved Ig group had better results than the other groups; however, we detected no statistically significant difference between the groups.

Pathologic features of MM like renal failure, thrombocytopenia, and eGFR of <60 mL/min were more often found in patients with suppressed uninvolved Ig, but statistical significance could not be found. Negative MM features such as anemia, stage-3 disease, osteolytic bone disease, hypercalcemia, LDH of >300 IU/L, BMPCs of ≥40%, and positive CRAB pathologies were more commonly seen in patients with suppressed uninvolved Ig (p<0.05). Positive responses to treatment (≥partial response and complete response) were more common in patients with preserved uninvolved Ig and revealed statistical significance (p<0.05).

Very few studies have investigated the prognostic importance of the preservation of uninvolved Igs. Some information comes from small series that included patients treated in the era before today’s novel agents [9,10]. Our study, which comprised 137 patients from a single center, is the first in Turkey on this subject. Kastritis et al. [8] showed the positive prognostic effects of preservation of uninvolved Igs on OS and disease-free survival. Better survival rates of patients with preserved uninvolved Ig levels were also found in our study, but a statistically significant difference was not observed. The most comprehensive study [8] on this issue included patients treated between 1990 and 2012, when novel agents were not being used effectively. Many of the patients of our study (75.9%) who were treated between 2003 and 2015 were treated with new-generation agents. Treating a large portion of the patients with new-generation agents could be the reason for the lack of statistically significant differences because this situation could eliminate the negative effects of uninvolved Ig suppression on survival. This result could highlight the necessity of new-generation chemotherapeutic drugs as first-line therapy for MM. Thus, unfavorable prognostic outcomes due to suppression of uninvolved Igs may be eliminated.

We found that higher levels of Ig were conserved in patients with a lower proportion of BMPC infiltration. There may be a positive relationship between normal plasma cell myeloma compartment and Ig level protection. It has been recognized that an extensive “abnormal” plasma cell population is associated with a high risk of progression in symptomatic disease in patients with asymptomatic myeloma or MGUS [11].

CRAB findings were not available in 11 of our patients, but these patients’ diseases had progressed rapidly and treatment was started. There was no suppression of uninvolved Ig in 3 of these patients, whereas the remaining 8 patients had uninvolved Ig suppression. CRAB pathologies were more common in myeloma patients with suppressed uninvolved Igs. Smoldering myeloma is known to form in a heterogeneous group of patients. Although the majority of patients have slow progression, some patients who are accepted as having early myeloma or CRAB-negative myeloma show an aggressive course. There is no molecular factor distinguishing these two groups, which are clinically and biologically different. The risk of progression is said to be associated with tumor burden. The fact that 28.8% of patients with smoldering myeloma are at high risk raises the issue of starting treatment early [12]. The presence of suppressed uninvolved Igs can be a clue to the necessity of starting treatment for patients who are diagnosed as having CRAB-negative myeloma.

## CONCLUSION

In conclusion, 13% of 137 patients with symptomatic MM had preserved uninvolved Ig levels. Patients with preserved uninvolved Igs had better response rates and pathologic findings but the preservation of the uninvolved Igs in patients was not an independent prognostic factor. In the same way, survival of patients with suppressed uninvolved Igs was shorter, but this could not be determined independently as a negative risk factor.

## Figures and Tables

**Table 1:**
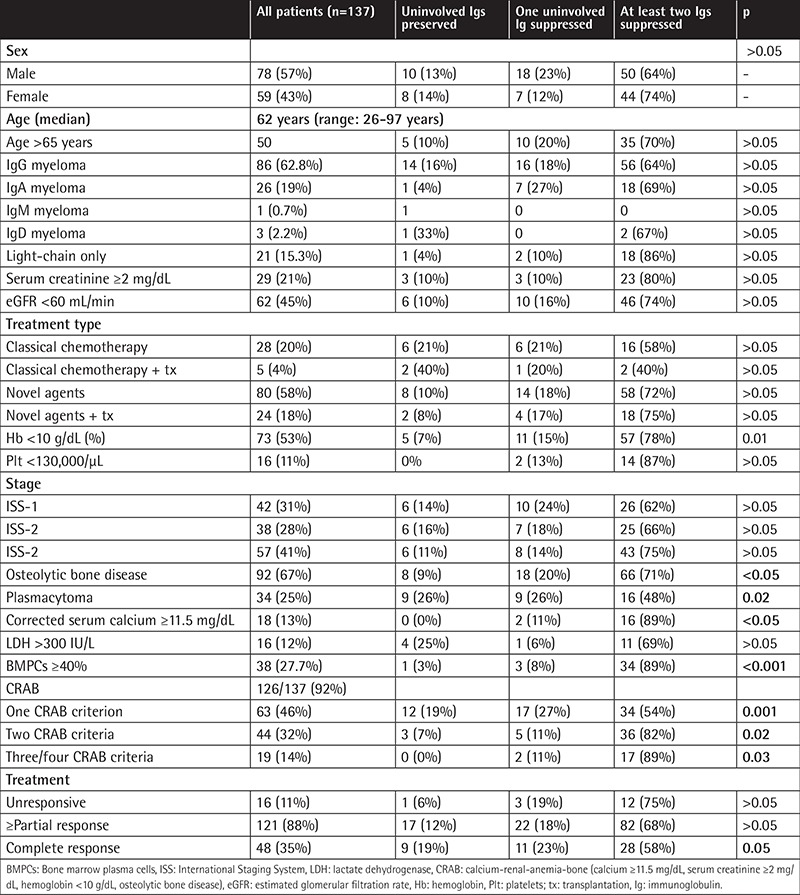
Characteristics of the patients in the analysis, of those with preserved immunoglobulins, those with suppression of one uninvolved immunoglobulin, and those with suppression of more than one uninvolved immunoglobulin.

**Table 2:**
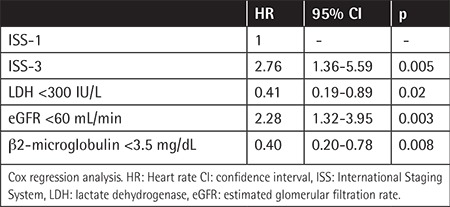
Multivariate analysis of factors associated with overall survival in 137 patients.
